# Influence of decision-making algorithms on the diagnostic accuracy using the current classification of periodontal diseases—a randomized controlled trial

**DOI:** 10.1007/s00784-023-05264-z

**Published:** 2023-09-27

**Authors:** Caspar Victor Bumm, Uta Christine Wölfle, Andreas Keßler, Nils Werner, Matthias Folwaczny

**Affiliations:** Department of Conservative Dentistry and Periodontology, University Hospital, LMU, Munich, DE Germany

**Keywords:** Dental education, Clinical decision-making, Periodontics, Decision trees

## Abstract

**Objectives:**

To examine the influence of the decision-making algorithms published by Tonetti and Sanz in 2019 on the diagnostic accuracy in two differently experienced groups of dental students using the current classification of periodontal diseases.

**Materials and methods:**

Eighty-three students of two different clinical experience levels were randomly allocated to control and study group, receiving the staging and grading matrix, resulting in four subgroups. All diagnosed two patient cases with corresponding periodontal charts, panoramic radiographs, and intraoral photographs. Both presented severe periodontal disease (stage III, grade C) but considerably differed in complexity and phenotype according to the current classification of periodontal diseases. Controls received the staging and grading matrix published within the classification, while study groups were additionally provided with decision-trees published by Tonetti and Sanz. Obtained data was analyzed using chi-square test, Spearman’s rank correlation, and logistic regression.

**Results:**

Using the algorithms significantly enhanced the diagnostic accuracy in staging (*p* = 0.001*, OR = 4.425) and grading (*p* < 0.001**, OR = 30.303) regardless of the clinical experience. In addition, even compared to the more experienced control, less experienced students using algorithms showed significantly higher accuracy in grading (*p* = 0.020*). No influence on the criteria extent could be observed comparing study groups to controls.

**Conclusion:**

The decision-making algorithms may enhance diagnostic accuracy in dental students using the current classification of periodontal diseases.

**Clinical relevance:**

The investigated decision-making algorithms significantly increased the diagnostic accuracy of differently experienced under graduated dental students and might be beneficial in periodontal education.

**Supplementary Information:**

The online version contains supplementary material available at 10.1007/s00784-023-05264-z.

## Introduction

The current classification of periodontal and peri-implant diseases and conditions published in 2018 [1] represents a major change in periodontology and shows numerous differences in comparison to the 1999 classification [2]. While the previous system distinguished between different types of periodontitis (chronic, aggressive), assuming different entities, the 2018 classification takes into account the scientific achievements of the last three decades, which ultimately could not prove any evidence of different etiology or pathogenesis in different conditions of periodontitis [2, 3]. The current classification further comprises a sophisticated system, in which different stages of severity are represented including complexity factors such as pocket probing depths and furcation involvement as well as predictive factors, which play a crucial role in the individual prognosis of periodontitis and the assessment of risk profiles [4]. Furthermore, the classification discriminates periodontal and gingival health from disease and distinguishes different periodontal conditions in patients with periodontitis [5, 6].

In order to implement the new classification and its various diagnostic tools into clinical practice and education in a simple yet cost-effective manner, Tonetti and Sanz [7] have published corresponding empiric decision-making algorithms that guide clinicians through the diagnostic process and support them in determining the correct diagnosis. Such decision trees have a flow-chart like structure and can be understood as inductive methods based on empirical data, which are a very popular tool not only in medicine, but also in machine learning and datamining [8]. They usually consist of a root node and internal nodes representing tests on attributes, branches that describe the test outcomes, and leaf nodes that represent the possible final decision.

To evaluate whether the decision-making algorithms published by Tonetti and Sanz [7] can facilitate the implementation of the current classification, the authors investigated their influence on the diagnostic accuracy in two groups of students with different clinical experience (in their first or last clinical year) in a randomized controlled trial.

## Materials and methods

### Ethical approval statement

The study was conducted in accordance with the ethical guidelines of the Helsinki Declaration and was approved by the Ethics Committees of the Medical Faculty of the Ludwig-Maximilians-University (No. 20–703).

### Study population

A total of *n* = 83 undergraduate students (10/2020–02/2020), *n* = 43 in their first clinical semester without previous clinical experience (1), and *n* = 40 in their third clinical semester with experience (2) at the Department of Conservative Dentistry and Periodontology, LMU, Munich, were included after verbal consent. All students were lectured equally by means of two separate regular teaching units (each of 45 min) about the current periodontal classification, whereas only students of group 2 have already gained clinical experience in diagnosing patients during practical courses. None of the students have, however, been introduced to the decision-making algorithms or received additional seminars on the classification. In both groups, students were randomly allocated to a control group (A) or a study group (B), resulting in the formation of four subgroups (A1 = control group less experienced, A2 = control group experienced; B1 = study group less experienced, B2 = study group experienced) in terms of simple randomization using the SPSS Software Program (SPSS Inc., Chicago, IL, USA). Both study groups were additionally introduced to the decision-making algorithms by Tonetti and Sanz by means of a single lecture of 45 min (B), resulting in the formation of four subgroups (Table 1, Supplement 1).

**Table 1:** Study group design

		Clinical experience	
		First clinical semester (1) *n* = 43	Third clinical semester (2) *n* = 40
Study design	Control group A: lectures, no decision-making algorithms	A(1): *n* = 21	A(2): *n* = 20
	Study group B: lectures and decision-making algorithms	B(1): *n* = 22	B(2): *n* = 20

### Clinical cases and sample diagnosis

Case documentation included general and specific anamnesis revealing neither smoking nor diabetes mellitus in any of the cases. Besides the corresponding periodontal charts containing pocket probing depths (PPD), gingival recessions (REC), clinical attachment levels (CAL), and bleeding on probing (BOP) at six sites per tooth as well as furcation involvements (FI), furcation degrees I–III [9], tooth mobility degrees I–III [10] and approximal plaque index (API) [11], panoramic radiographs, and intraoral photographs were supplied. Both cases were diagnosed generalized periodontitis stage III by all investigators in consensus as positive control using both the CAL and radiographic bone loss (RBL) criteria to create an unambiguous case (Table 2). Concerning primary criteria of grading both cases were classified grade C using indirect evidence of progression due to the absence of longitudinal data. Regarding extent, both cases presented with the same generalized (< 30% of teeth involved) high severity (CAL ≥ 5 mm, RBL extending to middle or apical third of the root and tooth loss ≤ 4 teeth) explaining same staging and extent. Case 2 (C2) presented with loss of 4 teeth. To avoid confusion among participants and since the number of teeth lost did not increase the severity as determined by CAL/RBL, no additional information was provided regrading reasons for tooth loss.

**Table 2:** Periodontitis stage (table based on Tonetti [5]) representation of the categories in italics for case 1, bold for case 2, and bold italics for the same category

Stage		I	II	III	IV
Severity	Interdental CAL	1–2 mm	3–4 mm	** ≥ *5 mm***	
	RBL	< 15%	15–33%	** > *33%***	
	Tooth loss due to periodontitis	*No tooth loss*		** ≤ 4**	≥ 5
Complexity		*Max PPD* ≤ *4 mm, mostly horizontal*	Max PPD $\ge 5$ mm, mostly horizontal	**In addition to stage II:** **PPD ≥ 6 mm, vertical bone loss > 3 mm, FB II–III, moderate ridge defect**	In addition to stage III: need for complex rehabilitation due to masticatory dysfunction, tooth mobility degree ≥ 2, bite collapse, drifting, flaring, less than 20 remaining teeth
Extent		Local		***Generalized***	

Concerning complexity, the first case (C1) showed a reduced periodontium with slight REC, only one site with PPD of 4 mm without BOP, and low corresponding CAL, whereas in the second case multiple sites with PPD ≥ 6 mm, REC, high CAL, and FI II-III could be observed. In terms of grading using indirect evidence of progression, both cases showed high bone loss/age ratios (> 1.0) consistent with rapid progression in the past and grade C. However, a considerable difference in case phenotype was noted. While the first case presented with perfect oral hygiene (API < 10%), e.g., destruction exceeded the given biofilm deposits, in the second case, proportionally with heavy biofilm accumulations (API = 78%), the severe periodontal destruction correlated (Tables 2, 3).

**Table 3:** Periodontitis grade of the presented cases (table based on Tonetti [5]) representation of the categories in italics for case 1, bold for case 2, and bold italics for the same category

Grade			A	B	C
Primary criteria	Direct evidence	Longitudinal radiographic data	No bone loss	Direct evidence	Longitudinal radiographic data
	Indirect evidence of progression	% bone loss/age	< 0.25	0.25–1.0	** > *1.0***
		Case phenotype	Heavy biofilm deposits with low levels of destruction	**Destruction commensurate with biofilm deposits**	*Destruction commensurate with biofilm deposits*
Grade modifiers	Risk factors	Smoking	***Non-smoker***	Smoker < 10 cigarettes/day	Smoker ≥ 10 cigarettes/day
		Diabetes	***No diabetes***	HbA1c < 7%	HbA1c ≥ 7%

### Experimental diagnosis

All groups diagnosed the 2 cases according to the current classification of periodontal diseases considering the main categories of diagnosis, i.e., extent, stage, and grade, solely using the provided materials under supervision of the investigators and were given a processing time of thirty minutes in total. Both control groups A(1) and A(2) diagnosed the cases exclusively with the staging and grading matrices that were published along with the classification [7, 12], whereas the study groups B(1) and B(2) received the staging and grading matrix and were additionally provided with the decision-making algorithms and a prior 45 min introduction to its application (Supplements 1).

### Questionnaire

A questionnaire was provided anonymously, on a voluntary basis, and in writing regarding questions in a 4-point Likert scale style including the questions: (1) Was the decision algorithm helpful in processing the cases? (2) In your opinion, is the decision algorithm easy to follow/well structured? (3) In your opinion, was the explanation of the decision algorithm in advance necessary for application, or understanding? (4) Would you use the decision algorithm when working on future cases? (5) In your opinion, should the decision algorithm be included in the curriculum?

### Statistical analysis

Statistical analysis was conducted using the SPSS Software Program (SPSS Inc., version 26, Chicago, IL, USA). The categorical variables (correct/incorrect diagnosis) for each diagnostic category (extent, stage, grade) of the four subgroups were given as relative amount for each subgroup. For univariate analysis, contingency tables were used with Pearson’s *χ*^2^ test. Linear correlation analysis has been done using the Spearman-Rho coefficient to determine interrelations between students’ experience, algorithm, and diagnosis regarding extent, staging, and grading. In order to determine the impact of the decision-making algorithms on the quality of diagnosis regarding the three categories, a bivariate logistic regression analysis was performed additionally, including 95% confidence intervals (95% CI), odds ratio (OR), and corresponding Wald test. Effect sizes (*f*) were calculated using Nagelkerkes *R*^2^ [13].

## Results

Chi-square test revealed significant (*p* = 0.001*) and highly significant (*p* < 0.001**) differences in diagnostic accuracy regarding the categories “staging” and “grading” in all compared groups when using the diagnostic algorithms (Table 4). In contrast, no significant differences could be observed in the category “extent.” In detail, comparing less experienced (1) with more experienced students (2), chi-square test showed a significantly higher diagnostic accuracy in staging (*p* = 0.001*) and grading (*p* < 0.001**) in more experienced students. Very similar to groups 1 and 2, the results of the study group (B) indicated highly significant increased diagnostic accuracy in staging (*p* < 0.001**) and grading (*p* < 0.001**) compared to controls (A). Furthermore, in direct comparison, less experienced students with decision-making algorithms B(1) showed highly significant differences in diagnostic accuracy in staging (*p* < 0.001**) and grading (*p* < 0.001**) when compared to their control A(1). In addition, even compared to the more experienced control A(2), less experienced students using the algorithms B(1) showed significantly higher accuracy in grading (*p* = 0.020*). Likewise, considering the more experienced groups A(2) and B(2), accuracy in staging (*p* = 0.026*) and in grading (*p* < 0.001**) was significantly higher in the study group B(2) than in the control A(2). Comparing cases, control groups A(1) and A(2) tended to underestimate the stage of C1 and to overestimate the stage of C2, while B(2) showed the largest consistency with the sample diagnoses. Further confirming these results, the evaluation of the additionally calculated Spearman’s rank correlation coefficient (Table 5) showed a significant correlation between the implementation of the algorithms and the diagnostic accuracy in the categories staging (ρ = 0.297; *p* < 0.001**) and grading (ρ = 0.504; *p* < 0.001**).

**Table 4:** Results of statistical analysis of correct answers by Pearson’s *χ*^2^ test (groups: A = control, B = study; (1) = less experienced, (2) = experienced)

	Extent			Staging			Grading		
Groups	Σ	%	*p*	Σ	%	*p*	Σ	%	*p*
(1)	64	74.4	0.207	47	54.7	0.001	52	60.5	< 0.001
(2)	66	82.5		63	78.8		68	85.0	
A	62	73.8	0.154	44	52.4	< 0.001	42	50.0	< 0.001
B	68	82.9		66	80.5		78	95.1	
A (1)	30	71.4	0.535 0.905 0.118	15	35.7	< 0.001 0.707 0.026	12	28.6	< 0.001 0.020 < 0.001
B (1)	34	77.3		35	79.5		40	90.9	
A (2)	32	76.2		29	69.0		30	71.4	
B (2)	34	89.5		34	89.5		38	100.0

**Table 5:** Results of statistical analysis of Spearman’s rank correlation coefficient ρ (*p*-value) (*Groups*: *A* = *c*ontrol, *B* = *study*; (*1*) = *less experienced*, (*2*) = *experienced*)

	Experience group A/B	Case	Algorithm group 1/2	Extent	Staging	Grading
Experience group A/B	1.000 (–)	0.000 (1.000)	− 0.370 (0.640)	0.098 (0.209)	0.255** (0.001)	0.274** (0.000)
Case		1.000 (–)	0.000 (1.000)	0.526** **(0.000**)	0.204** **(0.008)**	0.081 (0.301)
Algorithm group 1/2			1.000 (–)	0.111 (0.156)	0.297** **(0.000)**	0.504** (0.001)
Extent				1.000 (–)	0.305 (0.000)	0.262** (0.001)
Staging					1.000 (–)	0.270** (0.000)
Grading						1.000 (–)

The bolds highlight the statistically significant results

Additionally, calculated logistic regression analysis (Table 6) showed significant increased odds to achieve a correct overall diagnosis when more experienced (OR = 4.132; 95% CI = 1.862–9.174, ρ < 0.001) or even more when using the decision-making algorithms (OR = 11.905; 95% CI = 5.348–26.316; *p* < 0.001). Regarding staging, the chances are four times higher to obtain a correct diagnosis depending on algorithm (OR = 4.425; 95% CI = 2.119–9.259; *p* < 0.001) or experience (OR = 3.704; 95% CI = 1.776–7.752; *p* < 0.001).

**Table 6:** Results of bivariate logistic regression analysis of correct diagnosis

Variable	Modulator	OR (95% CI)	*p*-value	Effect size *f*	Chi-square (model) (*p*-value)
Correct diagnosis (all criteria)				0.620	< 0.0001
	Experience	4.132 (1.862–9.174)	< 0.001		
	Algorithmus	11.905 (5.348–26.316)	< 0.001		
Correct diagnosis (extent)				0.036	0.149
	Experience	1.664 (0.779–3.559)	0.188		
	Algorithmus	1.767 (0.827–3.774)	0.142		
Correct diagnosis (stage)				0.277	< 0.0001
	Experience	3.704 (1.776–7.752)	< 0.001		
	Algorithmus	4.425 (2.119–9.259)	< 0.001		
Correct diagnosis (grade)				0.934	< 0.0001
	Experience	6.993 (2.793–17.544)	< 0.001		
	Algorithmus	30.303 (5.076–100.000)	< 0.001		

In terms of grading, the chance to achieve the right diagnosis is even increased by the factor 30 (OR = 30.303; 95% CI = 5.076–100.000; ρ < 0.001), compared to experience by factor 7 (OR = 6.993; 95% CI = 2.793–17.544; ρ < 0.001). The correct diagnosis of the extent was estimated independent of experience (OR = 1.664; 95% CI = 0.779–3.559; *p* = 0.188) or algorithm (OR = 1.767; 95% CI = 0.827–3.774; *p* = 0.142). Taken as a whole, there is an advantage of the algorithm compared to experience in general diagnosis (*f* = 0.620; *p* < 0.0001), as well as the detail of staging (*f* = 0.277; *p* < 0.0001) and grading (*f* = 0.934; *p* < 0.0001), while the extension (*f* = 0.034; *p* = 0.149) remains unaffected.

**Fig. 1:**
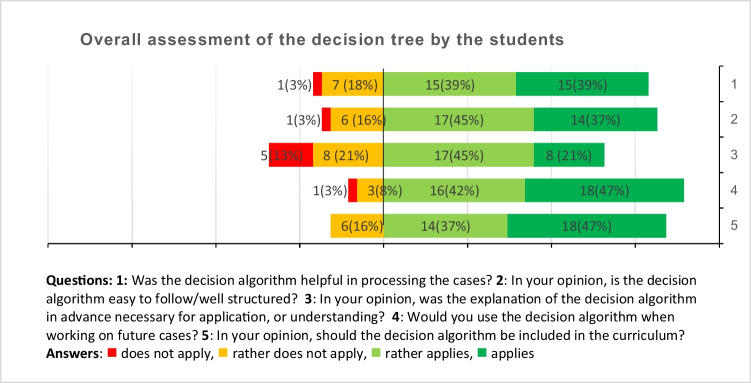
Results of the students’ questionnaire evaluation in total numbers and percentages

A total of 38 questionnaires have been entirely completed and were included into further analysis (Fig. 1). The overwhelming majority of replies reflected a positive appraisal of the diagnostic algorithm in terms of the additional value of the algorithm (78%), the comprehensibility of the decision tree (82%), the intention for future use (89%), and the recommendation for inclusion into the curriculum (84%).

## Discussion

The implementation of a new classification often poses a great challenge not only for clinical practice but also in education, especially if the previous classification has been valid for almost two decades [2]. Hence, establishing the classification of periodontal and peri-implant diseases must be seen as a process, the transition phase of which should be made as effective as possible, ensuring easy access and appliance for all potential users. The decision-making algorithms published by Tonetti and Sanz in 2019 [7] relating to the current classification should therefore be considered a component of this process.

Several research groups have already investigated the diagnostic accuracy of differently experienced clinicians using the current classification. Regarding consistency and accuracy among periodontal experts, general dentists, and undergraduate students, Marini et al. showed moderate consistency of the differently experienced examiners to the gold standard, while accuracy was almost perfect for staging and moderate for grading [14]. Interestingly, in this study, general dentists performed less well diagnosing the pre-selected cases when compared to undergraduate students and periodontal experts, especially regarding the grading. But also assessing the agreement exclusively among specialized periodontists using the current classification on nine severe periodontitis cases, Ravidà et al. demonstrated only a moderate concordance to a gold-standard diagnosis determined by periodontal experts involved in the development of the classification [15]. On the other hand, in a similar study to the one of Marini et al., Abrahamian et al. showed no significant differences regarding inter- and intra-rater reliability among postgraduate students, academics, and periodontal specialists, concluding that clinical experience is of less importance regarding the application of the classification [16]. None of these studies, however, investigated the decision-making algorithms that were published shortly after the classification by Tonetti and Sanz in 2019 [7] and intend to facilitate a simple and at the same time cost-effective implementation of the diagnostic modalities and nomenclatures in dental education and everyday clinical practice.

Therefore, the objective of this four-arm randomized controlled study was to evaluate the influence of these algorithms on diagnostic accuracy in differently experienced undergraduate dental students.

In general, the presented results provide evidence for the positive influence of these decision-making algorithms on the diagnostic quality and accuracy of differently experienced students showing highly significant differences especially in terms of staging and grading. In addition, subjective perceptions regarding helpfulness, understanding, and desire for implementation into the existing dental curriculum were overwhelmingly positive.

Comparison of less experienced students with more experienced showed significant differences in diagnostic accuracy considering staging and grading. This clearly verifies a difference between the two groups with regard to their experience and their basic knowledge of periodontal diagnostics, confirming the adequacy of the chosen cohorts.

Besides significant and highly significant differences between study groups and controls in both more and less experienced students, proving the advantages of the algorithms and their effectiveness alike, logistic regression analysis revealed a four times higher probability of a correct staging and an even 30 times higher chance for a correct grading when using the algorithms.

Concerning these results and the aforementioned under- and overestimation in staging compared to the sample diagnoses, an evaluation of the criteria of staging as proposed by the current classification seems reasonable. Herein, CAL is considered the primary criteria for staging. RBL on the other hand is recommended only secondary in the absence of measurable CAL or in mixed dentitions where detection of CAL is impaired [17, 18], since there is evidence for limitations such as low specificity that may result in “miss detection of mild and moderate periodontitis” when using RBL alone [17, 19]. Then again several limitations arise with CAL as well, mainly concerning the detectability of the cemento-enamel junction (CEJ) serving as reference for CAL measurement. While in healthy sites the gingival margin is located “approximately 1–3 mm coronal to the cemento-enamel junction” [20], gingival enlargement or swelling of the tissues at inflamed sites may lead to coronal advancement of the margin [20]. In case of gingival recessions, on the other hand, CEJ may be detected easier due to a more apical position of the marginal gingiva.

Besides these anatomical limitations regarding the reference point of CAL measurement, misdiagnosis might occur in subjects previously treated for periodontitis. It is commonly accepted that following successful conventional therapy of periodontal lesions, a long junctional epithelium is formed along the root surface leading to reduction of probing depths [21–25]. This reduction of probing depths is considered a main goal of periodontal therapy [26] and consequently results in reduction of CAL measurements.

Therefore, the sole use of CAL to describe severity of periodontal destruction may be misleading, resulting in under-estimation of actual loss of periodontal attachment in previously treated patients when using the current classification of periodontal disease.

These shortcomings of the solely use of CAL to determine periodontal stage may have led to the poorer results in accuracy of the control groups in the present study, considering that the matrix published within the classification primary recommends CAL to stage. Hence, especially the under-estimation of C1 that presented with low levels of CAL and at the same time high levels of RBL may be explained. On the other hand, the algorithms used by the study groups emphasize the primary use of RBL when initially classify a periodontitis case (step 1 [7]). In the following steps of staging (steps 2 and 3 [7]), CAL and RBL are considered equally by the decision-making algorithms, which might explain higher accuracy in staging by the study groups compared to their controls.

Furthermore, besides CAL or RBL, the role and relative importance of complexity of disease for the process of staging determined by probing depths, furcation status, tooth mobility, type of bone loss, extent of ridge defect, masticatory (dys)-function, and missing teeth or number of opposing pairs as proposed by the classification is not clarified by the staging matrix.

In contrast, the influence of complexity is embedded in the sequential structure of the decision-making algorithms, which might have facilitated the staging process in the study groups, bearing in mind that the cases differed considerably in complexity despite being diagnosed with the same stage.

Concerning grading, the results revealed an even higher spread when comparing diagnostic accuracy of the study groups and their controls. Again, the sequential structure of the algorithms seems to play a crucial role, since a weighting of the primary criteria as described in the classification cannot be withdrawn from the matrix, but from the algorithms. None of the clinical cases presented with a history of smoking or diabetes mellitus; therefore, grade modifiers were not applicable. Nevertheless, it has to be stated that following the algorithms clearly facilitates the process weighting the different criteria of grading.

Besides the advantages resulting from the use of the algorithms, it has to be highlighted that the significant differences in grading might also be explained by the fact that, compared to the former classification, grading is a completely new diagnostic modality, including thresholds of progression. While the severity of periodontal disease has already been described in the 1999 classification [2], grading and the corresponding criteria have been newly developed. However, the highly significant differences that were obtained in the present study impressively prove the value of the decision-making algorithms, being able to adopt new diagnostic processes even in less experienced clinicians with decent accuracies in periodontal diagnosis.

On the other hand, the decision-making algorithms seemed to have no influence on the users’ choice regarding the extent of periodontal disease. This finding is most likely to be justified by the fact that unlike the newly developed staging and grading system in terms of extent, only minor changes have been made to the 1999 classification.

Having compared the diagnostic accuracy of only two representative cases can be considered a limitation of this study, also because it examines the influence of the decision-making algorithms exclusively on the diagnosis of periodontitis, not including the other possible diagnoses of the current classification. A statement on the impact of the decision-making algorithms on the accuracy in detecting periodontal and peri-implant health [5, 27], gingivitis [28, 29], or peri-implant disease [17, 30] can therefore not be derived from this study. Despite statistically proven large effect sizes, a further limitation may be seen in the small sample size regarding students and two cases, as there was only limited time available to engage the services of the students during their regular clinical internship for the completion of the experimental diagnostic procedure.

Further research in lager scales and of differently experienced clinicians including general and specialized dentist is necessary to evaluate the decision-making algorithms as an everyday tool in periodontal patients.

## Conclusion

In case of severe periodontitis, regardless of the complexity or the case phenotype, the application of the investigated decision-making algorithms may significantly enhance the diagnostic accuracy in differently experienced dental students using the current classification of periodontal diseases.

In view of these results, the application of the algorithms may represent a promising approach for the implementation of the current classification of periodontal disease in dental education. Further research on the effectiveness of the algorithms, however, has to be accomplished to compose a potent recommendation for its regular use in dental educational institutions.

### Supplementary Information

Below is the link to the electronic supplementary material.

Supplementary file1 (DOCX 508 KB)

## Data Availability

The data that support the findings of this study are available from the corresponding author upon reasonable request.

## Abbreviations

BOP: Bleeding on probing
CAL: Clinical attachment level
CEJ: Cemento-enamel junction
CI: Confidence intervals
FI: Furcation involvement
OR: Odds ratio
PPD: Pocket probing depths
RBL: Radiographic bone loss
REC: Gingival recession
