# Supplementary figures and images for: Influence of decision-making algorithms on the diagnostic accuracy using the current classification of periodontal diseases—a randomized controlled trial
Source: Clin Oral Investig. 2023 Sep 27;27(11):6589–96. doi: 10.1007/s00784-023-05264-z (PMC10630190; doi:10.1007/s00784-023-05264-z)

Supplements


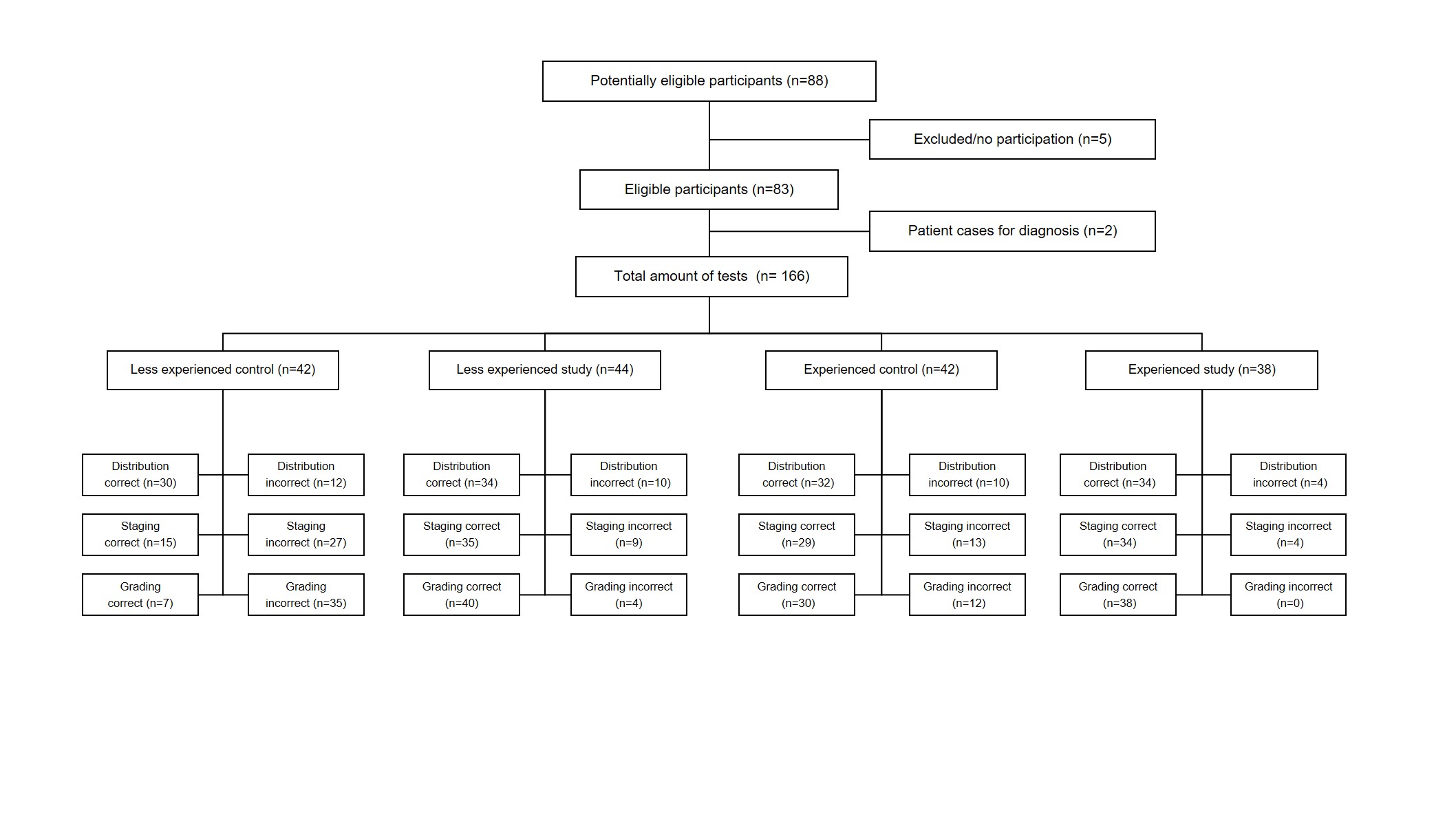
Supplement 1. Flow diagram of recruitment and results.

Supplement: Supplementary file 1 — Supplementary file1 (DOCX 508 KB) [file 784_2023_5264_MOESM1_ESM.docx]
